# Supplementary material for: Stacking Model‐Based Classifiers for Dealing With Multiple Sets of Noisy Labels
Source: Biom J. 2025 Mar 12;67(2):e70042. doi: 10.1002/bimj.70042 (PMC11898607; doi:10.1002/bimj.70042)
Supplement: Supplementary file 1 — Supporting Information [file BIMJ-67-e70042-s001.zip › density-based-ensemble-model-main/competitors_real_data/netlab-master/netlab-master/help/gmm.htm]

Netlab Reference Manual gmm


# gmm

## Purpose

Creates a Gaussian mixture model with specified architecture.

## Synopsis

```
mix = gmm(dim, ncentres, covartype)
mix = gmm(dim, ncentres, covartype, ppca_dim)
```

## Description

`mix = gmm(dim, ncentres, covartype)` takes
the dimension of the space `dim`, the number of centres in the
mixture model and the type of the mixture model, and returns a data
structure `mix`.
The mixture model type defines the covariance structure of each component
Gaussian:

```
  'spherical' = single variance parameter for each component: stored as a vector
  'diag' = diagonal matrix for each component: stored as rows of a matrix
  'full' = full matrix for each component: stored as 3d array
  'ppca' = probabilistic PCA: stored as principal components (in a 3d array
    and associated variances and off-subspace noise
```

`mix = gmm(dim, ncentres, covartype, ppca_dim)` also sets the dimension of
the PPCA sub-spaces: the default value is one.

The priors are initialised to equal values summing to one, and the covariances
are all the identity matrix (or equivalent). The centres are
initialised randomly from a zero mean unit variance Gaussian. This makes use
of the MATLAB function `randn` and so the seed for the random weight
initialisation can be set using `randn('state', s)` where `s` is the
state value.

The fields in `mix` are

```
  type = 'gmm'
  nin = the dimension of the space
  ncentres = number of mixture components
  covartype = string for type of variance model
  priors = mixing coefficients
  centres = means of Gaussians: stored as rows of a matrix
  covars = covariances of Gaussians
```

The additional fields for mixtures of PPCA are

```
  U = principal component subspaces
  lambda = in-space covariances: stored as rows of a matrix
```

The off-subspace noise is stored in `covars`.

## Example

```
mix = gmm(2, 4, 'spherical');
```

This creates a Gaussian mixture model with 4 components in 2 dimensions.
The covariance structure is a spherical model.

## See Also

`gmmpak`, `gmmunpak`, `gmmsamp`, `gmminit`, `gmmem`, `gmmactiv`, `gmmpost`, `gmmprob`

---

**Pages:**
Index


---

Copyright (c) Ian T Nabney (1996-9)
